# Multilevel exposure to adversity across the life course and biological implications among urban postpartum women: A cohort study protocol

**DOI:** 10.1371/journal.pone.0348413

**Published:** 2026-05-06

**Authors:** Anne Elizabeth Glassgow, Claire Flanigan, Anahi A. Gante Gaytan, Sage Kim, Aiman Soliman, Rachel Caskey, Almudena Veiga-Lopez, Irina A. Buhimschi, Amanda Knepper, Hagar Hallihan, Alicia Arredondo Eve, Sofija Jovanovic Gasovic, Sabrina Akter, Bridget Lawate, Zeynep Madak-Erdogan

**Affiliations:** 1 Department of Medicine, College of Medicine, University of Illinois Chicago, Chicago, Illinois, United States of America; 2 Department of Pediatrics, College of Medicine, University of Illinois Chicago, Chicago, Illinois, United States of America; 3 School of Public Health, University of Illinois Chicago, Chicago, Illinois, United States of America; 4 Institute for Genomic Biology, University of Illinois Urbana-Champaign, Urbana, Illinois, United States of America; 5 National Center for Supercomputing Applications, University of Illinois Urbana-Champaign, Urbana, Illinois, United States of America; 6 Department of Pathology, College of Medicine, University of Illinois Chicago, Chicago, Illinois, United States of America; 7 Department of Obstetrics & Gynecology, College of Medicine, University of Illinois Chicago, Chicago, Illinois, United States of America; 8 Department of Food Science and Human Nutrition, University of Illinois Urbana-Champaign, Urbana, Illinois, United States of America; PLOS: Public Library of Science, UNITED STATES OF AMERICA

## Abstract

Maternal mortality and morbidity in the United States remain substantially higher than in other high-income countries. Evidence suggests that exposure to chronic stress and social and environmental adversity contributes to maternal health risk through interconnected biological, psychological, and structural pathways. While these associations are documented, the molecular mechanisms linking adversity to maternal health outcomes remain poorly defined. This protocol describes a five-year, prospective, explanatory sequential mixed-methods cohort study (N = 200) designed to map how multilevel stress exposures influence biological and clinical outcomes among urban postpartum women. The study integrates geocoded neighborhood-level data, longitudinal electronic health records, and comprehensive interviewer-administered surveys assessing trauma, social support, and mental health. To identify the biological pathways of adversity, we employ multi-omics profiling of peripheral blood mononuclear cells—including DNA methylation, chromatin accessibility, and histone modification—alongside inflammatory and steroid hormone assays. Statistical frameworks, including Exposome-Wide Association Studies and spatial mediation modeling, will evaluate the interplay between socio-ecological stressors and molecular signatures during the postpartum period. Findings will advance understanding of the biological embedding of adversity and inform multilevel interventions to improve maternal health outcomes.

## Introduction

Maternal mortality and morbidity in the United States persist at higher levels than those of similarly resourced high-income nations, reflecting not only deficiencies in health care delivery but also broader social, environmental, and system factors that shape maternal health outcomes [1,2]^.^ In 2023, non-Hispanic Black women experienced a maternal mortality rate of 50.3 deaths per 100,000 live births, significantly higher than rates for non-Hispanic White (14.5/100,000), Hispanic (12.4), and non-Hispanic Asian (10.7) women, illustrating persistent differences in maternal outcomes [3]. The urgency of addressing maternal mortality is underscored by the temporal distribution of fatalities; specifically, nearly 53% of pregnancy-related deaths occur during the postpartum period, identifying the first year following childbirth as a uniquely critical window for life-threatening complications [3,4]. Data from multidisciplinary Maternal Mortality Review Committees indicate that 84% of pregnancy-related deaths are potentially preventable [5] underscoring the role of identifiable clinical, patient, and system-level factors amenable to targeted interventions.

The multifaceted maternal health crisis in the United States is driven by the convergence of evolving clinical complications and structural barriers. While hemorrhage and infection remain persistent complications, the clinical landscape has shifted over the last two decades. Cardiovascular conditions and behavioral health issues, specifically suicide and substance use, are now the leading causes of maternal death [5]^.^ Maternal health outcomes are shaped by structural barriers embedded within legal, economic, and geographic determinants that drive stark racial differences in maternal mortality. Specific structural determinants further worsen the crisis, ranging from the proliferation of obstetric care shortages to fragmented care transitions and the unstable “postpartum cliff” created by the loss of Medicaid coverage shortly after birth depending on the state’s participation in Medicaid expansion [6,7].

Accumulating evidence indicates that stress exposure is an important consideration in elucidating the biological, behavioral, and social pathways underlying maternal mortality, severe maternal morbidity, and overall maternal health across the perinatal period [8–10]. Stress exposure encompasses the experience of acute or chronic psychological, social, or environmental stressors, including traumatic events, and socioeconomic adversity, that activate neuroendocrine and inflammatory pathways and may influence physiological functioning during pregnancy [11–13]. While most research does not show a direct causal link between stress and maternal death, stress has been strongly associated with conditions that increase the risk of mortality and life-threatening maternal complications. Meta-analyses indicate that pregnant women exposed to stressful life events have a higher risk of preterm birth, low birth weight, and small for gestational age [14]^.^ Stress is a contributing risk factor that increases the likelihood of obstetric complications like hypertensive disorders, which are linked to maternal morbidity and mortality, and the development and exacerbation of mental health conditions and substance use, the leading cause of pregnancy-related deaths nationwide. Stress is an important risk factor for maternal health through interconnected biological, psychological, and social pathways; however, the precise mechanisms underlying these associations are poorly understood [15–18]. Structural stressors such as socioeconomic and neighborhood disadvantage become biologically embedded through neuroendocrine (e.g., the release of adrenaline in response to stress) and inflammatory (e.g., immune system activation) pathways, thereby increasing risk for maternal morbidity and mortality. Such integrated understanding can inform multilevel interventions, including stress-reduction and mental health strategies in clinical care alongside structural and policy approaches addressing upstream determinants of maternal health [19,20].

Advances in epigenomic technologies, quantitative assays, and computational pipelines that enable precise characterization of molecular regulation have not been applied to postpartum women [21]^.^ Such technology can provide insight into how social stressors and protective factors during the perinatal period shape epigenetic profiles and downstream maternal health outcomes [22,23]. This gap is especially consequential in the postpartum period, a window of heightened physiological and psychological vulnerability, where limited molecular data constrain our ability to understand risk [24].

Chicago represents an important setting to examine the impact of stress exposure on postpartum maternal health due to the racial inequities, socioeconomic disadvantage, and concentrated neighborhood-level violence and adversity, which are reflected in persistently elevated rates of maternal morbidity and mortality [8,25]. The biological embedding of stress via neuroendocrine and inflammatory pathways and its intersection with postpartum mental health remains insufficiently characterized in urban populations experiencing high cumulative stress burden [26,27].

To examine how stress and multilevel adversity influence biological and social epigenetic processes during the perinatal period, we are conducting a mixed-methods cohort study examining the physiological, mental health, and clinical consequences of stress and adversity among urban postpartum women. This protocol article describes the study design, conceptual framework, the collection of multimodal data, and the study implementation and recruitment status. This study will contribute to the understanding of mechanisms through which stress becomes biologically embedded during the postpartum period and contributes to maternal morbidity.

## Methods

### Study aims, design, conceptual model, setting, and research team training

#### Aims.

The Phase 1 study aims are to 1) describe the exposure to multilevel (neighborhood, family, and individual) adverse risk factors and 2) assess the effect of the multilevel exposures on biophysical outcomes. The Phase 2 aim is to 3) understand the multilevel context of daily experiences using qualitative photovoice methods among urban postpartum women.

#### Design.

This five-year prospective cohort study employs an explanatory sequential mixed-methods design, in which data are collected across two sequential phases. In Phase 1, the research team will collect and analyze quantitative data, and the results will be used to inform the development of Phase 2 qualitative data collection. The phased design ensures that qualitative findings further contextualize the quantitative results, and thus support a relevant socio-cultural interpretation rooted in the specific issues, needs, and experiences of underserved postpartum women residing in a common geographic area [28,29].

Study Aims 1 and 2 will be addressed in Phase 1, while Aim 3 is explored in Phase 2. Following the completion of Phase 2 data collection and analysis, findings from the quantitative and qualitative study phases will be considered in tandem to produce an integrated interpretation. A Community Advisory Board will serve as research partners, contributing to the interpretation and dissemination of study findings. This article describes the quantitative study protocol for Phase 1 of the study.

#### Conceptual model.

**Fig 1:**
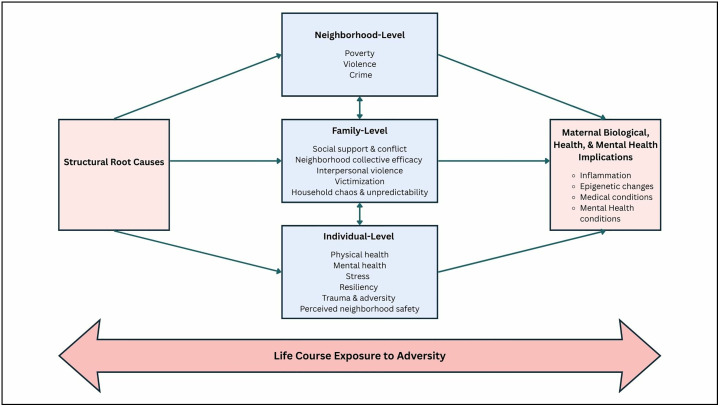
Socioecological-Biological Model of exposure to structural root causes and maternal biological, health, and mental health implications and outcomes.

Fig 1 displays the Socioecological-Biological Model of exposure to structural root causes and the resultant maternal physical and mental health outcomes that guide the study. Structural root causes produce adversity by generating unequal exposure to chronic stress at multiple levels (i.e., neighborhood, family, and individual) across the life course, resulting in deleterious biophysical outcomes such as an increase in inflammation, chronic medical conditions, and harmful epigenetic changes.

#### Setting.

A transdisciplinary research team representing twelve academic disciplines will conduct the research at the University of Illinois Chicago (UIC)/University of Illinois Hospital and Health Sciences System (UI Health). During Phase 1 quantitative data collection, two hundred postpartum women are being recruited for the study. UI Health primarily serves Chicago’s South and West Sides, community areas that are characterized by socioeconomic barriers that correlate with disproportionately poor health relative to other areas in the city. Approximately 85% of pregnant and postpartum patients who receive care at UI Health identify as Black or Hispanic, and approximately 75% are publicly insured through Medicaid.

#### Research team training.

During the study planning phase, standard operating procedures were developed to standardize guidelines for clinical tasks (e.g., blood and urine collection); manage medical and non-medical emergencies (e.g., vasovagal reactions or participant distress); collect, process, and store biological samples (e.g., sample collection, processing, and storage). Additionally, a master study flow document detailing the order of operations was developed, and the questionnaire was created and piloted. The Principal Investigator conducted comprehensive training on protocol adherence and data collection, integrity, security, and quality. The research team members involved with data collection received intensive training on the collection of sensitive data such as trauma history, mental health, and neighborhood conditions. The research team conducted several mock visits and simulated interactions with participants under the guidance of perinatal social workers. These simulations serve as a critical feedback mechanism, allowing the research team to refine the study flow, practice administration of the survey instrument, optimize the overall approach to participant engagement, and recognize and respond to participant distress.

### Participant recruitment, enrollment, data collection, and ethics approval

#### Recruitment.

The inclusion criteria include women who are postpartum (delivered a live infant within the past 12 months), English-speaking, 18 years or older, delivered at and receive medical care at UI Health, and have the capacity to provide informed consent. The UIC Center for Clinical and Translational Science Clinical Research Data Warehouse supplies the research team monthly with a list of potentially eligible participants. The list is compiled via the UI Health electronic health record (EHR) in accordance with the study inclusion criteria.

Study recruitment began in July 2025 and is proceeding on a rolling basis. Participant recruitment and quantitative data collection are expected to be completed by March 2027, with data analysis and qualitative data collection extending through 2028. The research team utilizes a standardized set of technologies to manage participant recruitment and scheduling. The research team documents recruitment activities in a Research Electronic Data Capture (REDCap) database, a secure web-based platform, and utilizes Zoom Phone for recruitment calls and text messages. To ensure data security, all text-based correspondence is strictly limited to non-sensitive, non-protected health information content. Scheduling is managed through a study-specific, de-identified institutional email account (i.e., UIC Outlook). The research team contacts eligible participants from the list by email, text, and phone call to explain the study, confirm eligibility, and schedule an in-person appointment. Once an eligible participant schedules an appointment, the research team uses the study email account to send a calendar invitation and appointment reminders. To maintain confidentiality, email communications do not contain protected health information.

#### Enrollment.

Enrollment and the study visit occur at the UIC Maternal Health Postpartum Laboratory. All participants complete a written informed consent process prior to engaging in any study activities. In accordance with trauma-informed research practices, the research team is trained to create and maintain a safe and comfortable physical environment, facilitating participant comfort by providing comfortable seating options, water, snacks, and optional childcare. To ensure participant comfort, water and snacks are offered upon arrival. The research team then provides the potential participant with a copy of the study consent form, reviews each page to ensure understanding, and frequently pauses at intervals to answer questions. If the potential participant agrees to participate, she electronically signs a REDCap E-Consent form on a study tablet. Participants who decline to participate are thanked for their time, provided with parking validation if needed, and escorted to the lobby.

#### Data collection.

Following enrollment, a 133-question survey, developed specifically for this study, is administered to all participants (Table 1). Throughout the survey, the research team reminds participants that they may decline to answer any question. After completion of the survey, participants’ biological samples (i.e., blood and urine) are collected. Participants provide a urine sample, and a certified phlebotomist performs a venous blood draw under sterile conditions while participants remain seated or supine to prevent dizziness or fainting. After data collection, participants are provided with postpartum medical and mental health resources, offered free Narcan and fentanyl test strips, compensated for their time and participation in the study ($125), and receive parking validation if needed. The data collection appointment takes an average of 60–90 minutes to complete.

**Table 1:** Psychosocial and Contextual Measures Collected via Interviewer-Administered Questionnaires.

Construct	Instrument (Source)	Validation Status	Scoring Interpretation
**Demographics**	Basic Demographics	Research Team- Developed	Not scored; descriptive demographic variables
	The American Community Survey [30]	Validated (modified)	
	Statistical Policy Directive No. 15 [31]	Validated (unmodified)	
	Birthplace (PhenX 10201) [32]	Validated (unmodified)	
	Educational Attainment – Individual (PhenX 11002) [33]	Validated (unmodified)	
**Social Health**	Protocol for Responding to and Assessing Patients’ Assets, Risks and Experiences	Validated (modified)	Cumulative number of distinct risks that are present vs. absent
	Hunger Vital Sign [34]	Validated (unmodified)	Higher scores indicate greater risk for food insecurity.
**Community & Social Environment**	Neighborhood Collective Efficacy – Community Cohesion and Informal Social Control (PhenX 210801) [35]	Validated (modified)	Higher scores indicate greater community cohesion.
	Neighborhood Safety (PhenX PX210901) [36]	Validated (unmodified)	Lower scores indicate greater perceived safety.
	Household Chaos and Unpredictability (PhenX 300501) [37]	Validated (modified)	Higher scores indicate greater household chaos.
	Neighborhood Description Scale	Research Team- Developed	Higher scores indicate lower perceived neighborhood amenities and upkeep.
	Multidimensional Scale of Perceived Social Support [38]	Validated (unmodified)	Higher scores indicate greater perceived social support.
**Social Adversity & Trauma**	Adverse Childhood Experience Questionnaire for Adults [39]	Validated (unmodified)	Higher scores indicate exposure to a greater number of adverse childhood experiences.
	Humiliation, Afraid, Rape, and Kick questionnaire [40]	Validated (unmodified)	Score ≥1 indicates exposure to intimate partner violence.
	Everyday Discrimination Scale [41]	Validated (modified)	A higher total score indicates a greater frequency of adverse experiences.
	Primary Care PTSD Screen for DSM-5 [42]	Validated (unmodified)	Score ≥3 indicates probable post-traumatic stress disorder (positive screen).
**Mental Health & Wellbeing**	Patient-Reported Outcomes Measurement Information System Scale V1.2 [43]	Validated (modified)	Higher scores indicate better perceived overall health.
	Sleep Quality Scale	Research Team- Developed	Higher scores indicate better perceived sleep quality.
	Perceived Stress Scale (Phen X 180801) [44]	Validated (unmodified)	Higher scores indicate greater perceived stress.
	Prenatal and Postpartum Depression (PhenX 241401) [45]	Validated (unmodified)	Score ≥10 indicates depressive symptoms (positive screen).
	Generalized Anxiety Disorder Screener [46]	Validated (unmodified)	Score ≥10 indicates anxiety symptoms (positive screen).
	Brief Resilience Scale [47]	Validated (unmodified)	Higher scores indicate greater resilience and ability to recover from stress.
**Substance Use**	Tobacco, Alcohol, Prescription Medication, and Other Substance use, first-stage screening component-1 [48]	Validated (unmodified)	Monthly use (tobacco/alcohol) or any use (illicit/prescription misuse) indicates positive screening.
	National Institute of Drug Abuse-Modified ASSIST V2.0 [49]	Validated (modified)	Any response other than “never” indicates at-risk use.

#### Ethics approval.

All study materials, protocols, and processes were approved by the UIC Office for the Protection of Research Subjects Institutional Review Board (2023−0624).

### Outcomes

#### Psychosocial self-reported data.

The research team created a 133-item interviewer administered questionnaire to collect demographics and to measure constructs that include neighborhood conditions and experiences, family relationships, household conditions, social support, relationships, mental health, stress, resilience, trauma experiences, violence exposure, substance use, global health, and sleep quality. Participant neighborhood-level data (e.g., social, economic, and environmental conditions) will be appended using patient residential address. Neighborhood variables are coming from various publicly available data sources (e.g., United States Census Bureau, American Community Survey, Cook County Medical Examiner’s Archive, Chicago Health Atlas, Chicago Data Portal). Table 1 describes the measurement of psychosocial constructs.

#### Biological markers data.

We are collecting biological data from blood and urine. Plasma concentrations of pro-inflammatory cytokines, including Interleukin-6 and Interferon-gamma, and chemokines, including C-X-C Motif Chemokine Ligands 1 and 9 and C-C Motif Chemokine Ligands 4 and 23 are measured by enzyme-linked immunosorbent assay. Additional inflammatory markers include C-Reactive Protein, Extracellular Newly Identified RAGE-Binding Protein, Matrix Metalloproteinase-7, and A Disintegrin and Metalloproteinase with Thrombospondin Motifs. Growth hormone also is measured given its immunomodulatory properties and role in metabolic and immune interaction [50]. Plasma vimentin, a structural protein released extracellularly by activated immune cells under inflammatory conditions [51], is being quantified. Serum 25-hydroxyvitamin D are measured as a marker of nutritional and immune status, given its established role in modulating innate and adaptive immunity [52]. Metabolomic profiling is conducted using gas chromatography–mass spectrometry to characterize a panel of approximately 40 steroid hormones, including cortisol and estradiol, providing insight into hypothalamic-pituitary-adrenal axis activity and broader endocrine function during the postpartum period. Epigenomic analyses are performed on peripheral blood mononuclear cells (PBMCs) and will include Reduced Representation Bisulfite Sequencing to assess genome-wide DNA methylation patterns, Assay for Transposase-Accessible Chromatin with sequencing to map chromatin accessibility, and Cleavage Under Targets and Release Using Nuclease to evaluate histone modifications and glucocorticoid receptor binding. Together, this multi-omics approach integrating proteomics, metabolomics, and epigenomics will enable the identification of biological signatures and mechanistic pathways linking chronic stress exposures to maternal health outcomes. Plasma is processed and stored at −80°C, whereas PBMCs are kept in liquid nitrogen for long-term stability. Urine samples also are stored at −80°C and will be analyzed for exposure to heavy metals and per- and polyfluoroalkyl substances.

#### Clinical data.

The research team will abstract clinical data from the UI Health EHR for all consented participants. The EHR data abstraction will include two years prior to delivery through 12 months postpartum to capture clinical data prior, during, and post pregnancy. Data will include healthcare utilization (e.g., number of prenatal and postpartum visits, hospitalizations, and emergency department visits); laboratory results (e.g., hemoglobin A1c, complete blood counts, glycosylated hemoglobin, and comprehensive metabolic panel); vital signs, biometric measures, physical health conditions (e.g., high blood pressure and diabetes); mental health conditions (e.g., depression and anxiety); and birth outcomes and indicators (e.g., gestational age, birth weight, mode of delivery).

### Biospecimen handling and data management and protection

Data will be collected in a private area and entered directly into a Health Insurance Portability and Accountability Act (HIPAA)-compliant REDCap database. Biospecimens will be immediately labeled with a unique, study-specific identification number and the collection date and will not contain personal identifiers. On the day of collection, samples are transported to the UIC Department of Pathology Laboratory for processing within 4 hours of collection to obtain plasma, serum, and PBMCs, which are isolated by centrifugation, and then stored in a locked −80 °C freezer within the same laboratory. Every two months, the processed biospecimen samples are transported to the University of Illinois Urbana-Champaign for analysis; dry ice is used to preserve cold chain integrity and sample viability during transport. All data are being stored on the Nightingale computing cluster at the National Center for Supercomputing Applications at the University of Illinois, which is a high-performance computer cluster for sensitive data that operates as a HIPAA-compliant and secure system. The research team will preserve all sequencing data, secondary datasets, and associated codes in public repositories with a Digital Object Identifier. All finalized data will be stored and archived in the Illinois Data Bank; a public-access, file-based repository specifically designed to centralize, preserve, and provide reliable access to de-identified research data. All finalized data will be uploaded to the National Center for Biotechnology Information Gene Expression Omnibus repositories, in accordance with the National Institutes of Health Data Management and Sharing Policy.

Loss of confidentiality is mitigated through multiple safeguards. Authorized study personnel have access to identifiable participant information during recruitment and data collection; all data are subsequently de-identified using unique study identifiers prior to analysis. All data will be entered directly into a HIPAA-compliant REDCap database, de-identified using unique study codes, and stored on secure institutional servers. Electronic health record abstractions will be performed within UIC’s encrypted environment and only authorized research personnel will have access. Identifiers will be removed prior to data analysis and any public data sharing. All data will be de-identified before analysis. Biospecimens and sequencing data will be deposited only after institutional review and archiving, ensuring privacy protection.

### Statistical analyses

Throughout, descriptive and bivariable statistics will follow standard approaches. Continuous variables will be presented as means with standard deviations or medians with interquartile ranges, while categorical variables will be summarized as frequencies and percentages. Two broad categories of analysis will be conducted for Aims 1 and 2. We will begin by defining the geographic footprint for each participant within the city of Chicago based on their history of home and work addresses, which is required to calculate aggregated exposure metrics related to environmental exposures and social risks. Neighborhood indices quantify social conditions, environmental pollution and hazards, and safety exposures, which will be appended to participant-level data using the patient’s address. The aggregated exposure for each participant will be joined to the biological metrics. Next, we will examine associations between exposures and biological outcomes within an Exposome-Wide Association Study framework using generalized linear models. Plasma steroid and biomarker concentrations will be evaluated using linear regression with Bonferroni or Sidak corrections; non-parametric Kruskal-Wallis and Dunn post hoc tests will be applied when distributional assumptions are not met. To further understand the mechanistic pathways through which neighborhood context may influence maternal health, we will conduct a set of mediation analysis to assess the paths between stress exposure, changes in biomarkers and maternal outcomes. Epigenomic sequencing data, including DNA methylation profiles, chromatin accessibility, and glucocorticoid receptor binding patterns derived from PBMCs, will be modeled using spatial regression to predict variation in epigenetic markers across multiscale exposures (i.e., Assay for Transposase-Accessible Chromatin with sequencing and spatial Cleavage Under Targets and Release Using Nuclease). A total of 200 postpartum participants across at least 40 census blocks provides 90% power to detect a medium effect size at 0.5 at α = 0.05, assuming an intraclass correlation of 0.05.

For Aim 2, data analysis will focus on evaluating spatial patterns of maternal health outcomes and self-reported survey data in relation to exposure to neighborhood-level structural root causes rather than individual-level risk alone. We will begin by assessing the extent to which maternal risk factors related to neighborhood context explain variation in health outcomes beyond idiosyncratic risk at the individual and family levels by assessing spatial autocorrelation in maternal health outcome variables collected via psychosocial self-reported data, clinical electronic health records, and biological markers. We will calculate relative distances among study participants, and the distance matrix will be joined with the survey data, clinical electronic health records, and biomarkers to conduct the spatial autocorrelation analysis. Findings from this aim will identify the degree to which neighborhood-level exposures contribute to disparities in maternal biological and health outcomes and will inform the development of place-based strategies to address structural determinants of postpartum health.

### Study update and discussion

As of March 11, 2026, 101 participants have been enrolled in the study and completed data collection. The study offers several advantages and valuable contributions to maternal health research. This study employs an explanatory sequential mixed methods design, consisting of an initial quantitative phase followed by a qualitative phase to further explain and contextualize the numerical findings. By integrating these methods, the study achieves greater interpretive depth, transforming abstract statistical outputs into contextually grounded conclusions. In the context of maternal health, this design is particularly valuable because it moves beyond clinical metrics to capture the living experiences of perinatal women. This focus is critical given that the study is enrolling a postpartum population that has high exposure to stressors and poor health outcomes [26,53]. The study predominantly enrolls Black and Hispanic women residing in Chicago’s West and South Side neighborhoods, where systemic poverty and violence manifest as toxic stress, resulting in a cascade of adverse maternal health outcomes [27,54,55]. While disadvantage is often linked to poor maternal health, the underlying and intersecting mechanisms and pathways by which social and environmental exposures affect maternal health are largely unknown.

Significant investments have been made to develop technologies, standardize reagents for quantitative measurements, and establish computational pipelines to analyze and interpret epigenetic data. Despite these advances, few studies have leveraged genetic and epigenetic data from at-risk populations, especially among perinatal women, to examine how social stressors and protective factors drive the molecular changes underlying health outcomes. Investigating these intricate pathways requires significant statistical power; accordingly, the study’s sample size of 200 participants is notably large for studies involving the collection and analysis of biological specimens [56–58]. Biomarker and molecular epidemiological studies are frequently constrained by the logistical complexity, cost, and participant burden associated with biological sample collection and processing, often resulting in small sample sizes that necessitate careful statistical optimization to maximize the information extracted from limited biological specimens [59]. More broadly, low statistical power has been identified as a pervasive problem across the biological and neurosciences, with median power estimated at only 8–31%, driven largely by small sample sizes [60].

The larger sample size provides substantially greater statistical power to detect meaningful effects and reduces the risk of inflated effect sizes and irreproducible findings. Another strength of our approach is the multi-dimensional nature of our biological data, encompassing the Cleavage Under Targets and Release Using Nuclease method for histone profiling, a steroid hormone panel, inflammatory cytokines, DNA methylation, and DNA accessibility. Multi-omics and multi-layered epigenomic approaches are increasingly recognized as essential for capturing the complexity of biological regulation that single-assay designs cannot achieve [61]. Importantly, by collecting these diverse molecular data from a cohort of this size, we are well-positioned to identify environmental factors that produce coordinated, dose-dependent biological signatures across multiple systems. For example, graded exposure to neighborhood violence may manifest as a dose-dependent increase in cortisol that, in turn, drives downstream changes in inflammatory cytokine levels, DNA methylation, and shifts in chromatin accessibility, which is consistent with the biological embedding model [62–65]. Our design allows us to trace these cascading/interconnected effects across biological layers within the same individuals, enhancing our capacity to identify mechanistic pathways linking environmental exposures to molecular outcomes.

This study has several limitations that should be acknowledged. A key limitation of this study is its cross-sectional design; because survey and biological data are collected at a single time point, the results provide only a snapshot of the population. This allows for the identification of correlations but precludes drawing conclusions about causality, trends, or potential timing biases. To address this, future research should employ longitudinal designs to track changes over time and establish clearer causal relationships between exposures, biological markers, and clinical outcomes.

Another limitation is that measuring individual exposures based on the history of home and work locations is subject to uncertainty compared to the use of travel diaries and wearable devices. Utilizing spatially weighted kernels centered on home and work addresses, combined with mobility information from self-reported survey data, can potentially attenuate this limitation and provide a more realistic measure of participants’ mobility, thereby improving the quantification of their exposure to different factors. We also plan to assess the sensitivity of the findings to changes in spatial weighting to quantify uncertainty ranges related to specific exposure factors.

Biomarker variability related to physiological stage and ancestry may influence study findings. Previous work identified a ten-cytokine blood signature for depression in postmenopausal women, with four cytokines differing among Black pregnant women. These biomarker patterns may differ in postpartum women due to hormonal changes. Coupling cytokine detection with epigenetic and other molecular data will help identify novel biomarkers of maternal health. Additionally, genetic ancestry and ethnicity will be considered as factors that may influence baseline levels of blood biomarkers.

Despite the limitations, this study will generate comprehensive multilevel data, spanning clinical, biological, and socio-environmental factors, from postpartum women living in high-risk, low-resource communities. By utilizing a multi-omics approach with a robust sample size of 200 participants, this study sets a new methodological benchmark for capturing how social stressors are embedded biologically. It moves the field beyond isolated biomarker analysis to a more integrated systems biology model, tracing how environmental toxins and systemic stressors create cascading molecular signatures across histone profiling, DNA methylation, and inflammatory pathways. The findings could provide the biological evidence needed to advocate for targeted investments in high-risk neighborhoods. By demonstrating a dose-dependent relationship between neighborhood violence and molecular health markers, the research supports policies that treat urban poverty and systemic violence as public health crises requiring structural intervention to improve long-term maternal outcomes. For health providers, this study has the potential to highlight the necessity of trauma-informed perinatal care that accounts for a patient’s living environment as much as their clinical metrics. Finally, in collaboration with our Community Advisory Board and community partners, we are committed to transparency as we share our findings with maternal health stakeholders through academic publications, professional conferences, and direct community engagement and outreach.
